# New Hampshire Medical Society

**Published:** 1882-12-20

**Authors:** 


### *NEW HAMPSHIRE MEDICAL SOCIETY.*

A copy of the Transactions of the New Hampshire Medical Society, held in Concord, June, 1881, has been kindly sent us by the Secretary, Dr. G. P. Conn. It is a neat volume of 167 octavo pages.

It contains a number of able and interesting papers. First, the address of the President, Dr. G. P. Conn, on "State Medicines."

Report on Surgery—Dr. A. H. Crosby.

Cases of Malignant Disease, with a Report on the use of the Dermal Curette—Dr. F. A. Stillings.

A Contribution to the Study of Fractures and Dislocations—Dr. J. R. Ham.

Puerperal Convulsions—Dr. J. W. Parsons.

Conservatism in the Practice of Medicine—Dr. J. R. Kimball.

Hypodermic Medication—Dr. M. H. Felt.

Nihilism in Medicine—Dr. D. W. Jones.

Death: Its Physical Aspect—Dr. A. Richardson, and Reports of District Societies.

The officers elected for the ensuing year are—

*President*—Dr. H. B. Fowler. *Vice-President*—Dr. A. H. Crosby. *Secretary*—Dr. G. P. Conn. *Treasurer*—Dr. D. S. Adams.

